# Theme trends and knowledge structure on choroidal neovascularization: a quantitative and co-word analysis

**DOI:** 10.1186/s12886-018-0752-z

**Published:** 2018-04-03

**Authors:** Fangkun Zhao, Bei Shi, Ruixin Liu, Wenkai Zhou, Dong Shi, Jinsong Zhang

**Affiliations:** 1 Department of Ophthalmology, The Fourth Affiliated Hospital of China Medical University, Shenyang, China; 2 Eye hospital of China Medical University, Shenyang, China; 3 Key Lens Research Laboratory of Liaoning Province, Shenyang, China; 4 Department of Physiology, China Medical University, Shenyang, China; 5 Department of Ophthalmology, Second People’s Hospital of Fuxin City, Fuxin, China

**Keywords:** Choroidal neovascularization, Bibliometric analysis, Co-word analysis, Social network analysis

## Abstract

**Background:**

The distribution pattern and knowledge structure of choroidal neovascularization (CNV) was surveyed based on literatures in PubMed.

**Methods:**

Published scientific papers about CNV were retrieved from Jan 1st, 2012 to May 31st, 2017. Extracted MeSH terms were analyzed quantitatively by using Bibliographic Item Co-Occurrence Matrix Builder (BICOMB) and high-frequency MeSH terms were identified. Hierarchical cluster analysis was conducted by SPSS 19.0 according to the MeSH term-source article matrix. High-frequency MeSH terms co-occurrence matrix was constructed to support strategic diagram and social network analysis (SNA).

**Results:**

According to the searching strategy, all together 2366 papers were included, and the number of annual papers changed slightly from Jan 1st, 2012 to May 31st, 2017. Among all the extracted MeSH terms, 44 high-frequency MeSH terms were identified and hotspots were clustered into 6 categories. In the strategic diagram, clinical drug therapy, pathology and diagnosis related researches of CNV were well developed. In contrast, the metabolism, etiology, complications, prevention and control of CNV in animal models, and genetics related researches of CNV were relatively immature, which offers potential research space for future study. As for the SNA result, the position status of each component was described by the centrality values.

**Conclusions:**

The studies on CNV are relatively divergent and the 6 research categories concluded from this study could reflect the publication trends on CNV to some extent. By providing a quantitative bibliometric research across a 5-year span, it could help to depict an overall command of the latest topics and provide some hints for researchers when launching new projects.

## Background

Choroidal neovascularization (CNV) is defined as a process of blood vessel growth abnormality from the choroid layer into the retina layer [1] and this symptom could result in a sudden deterioration of central vision, metamorphopsia, or even worse, hemorrhage of new blood vessels. CNV formation is the pathological termination in a set of chorioretinal diseases, such as age-related macular degeneration (AMD), pathological myopia, polypoidal choroidal vasculopathy (PCV) [2]. The pathogenic mechanism of CNV development is yet not well understood. However, studies have shown that vascular endothelium growth factor (VEGF) plays an essential role in the development of CNV [3]. Anti-VEGF agents are proved to be useful for improving the clinical outcome of wet AMD. However, CNV membranes cannot subside completely after anti-VEGF intravitreal injections, and visual acuity can be enhanced in only 30–40% of the patients after treatment [4].

Bibliometry is used to make quantitative analysis and decipher the hot topics of literatures. Hence, bibliometry is helpful for scientists to monitor the growth and patterns of a specific scientific field. Methods such as co-citation analysis and co-word analysis can be used to reveal the hot topics of researches [5]. Co-word analysis is a content analysis method based on the principle that a selected literature can be represented by a set of professional words [6]. In this analysis, the relationship of two interested professional words is defined by the frequency of their co-existence in the same article. This relationship is then used to deduce the research focus and framework by categorizing the words into different areas using statistical analyses including cluster analysis, factor analysis and multidimensional scaling analysis. In a field of interest, cluster analysis has been adapted comprehensively to acquire the research themes [7].

Hierarchical cluster analysis is a widely used classification technique in many scientific areas. With this hierarchical cluster analysis, using a grouping algorithm and similarity measurement, a dendrogram can be generated and clusters can be categorized [8]. The density and centrality of each cluster can be calculated according to the result of the hierarchical cluster analysis. In addition, a strategic diagram was applied to interpret the tendency of these clusters.

Social network analysis (SNA) is a method to study the relationship among a set of factors and to analyze the connections with regard to network theory that includes nodes (representing extracted MeSH terms in this study) and ties (representing the relationship of these MeSH terms in this study) [9]. In intricate networks, recognizing the impactful nodes is of great theoretical and practical importance. Centrality measurement is an important measuring method used for analyzing networks, and degree, betweenness and closeness centrality are the three most widely accepted indexes which are established to compare the centrality of nodes in networks [10]. A node’s degree centrality is the number of direct links it has with other nodes in the network, which can reflect how important that node is to the network to some extent. Betweenness measures the influence of a given node in a network. It is calculated as how frequently a node lies on the geodesic paths of other nodes in the network. Closeness centrality is defined as the inverse sum of shortest distances from a node to all other nodes, which means the higher closeness centrality is, the closer the node to the others [11].

Aiming to provide an intuitional knowledge structure in the bibliometric perspective for future researchers, we tried, for the first time, to have a quantitative analysis of the research characteristics and popular topics in a wider field of “CNV”. In this study, cluster analysis based on MeSH terms co-occurrence and strategic diagram were used to provide a picture of the research status and emerging issues of CNV in retinal disease. We also applied SNA analysis to provide a visible knowledge structure of the relationship between CNV and its etiology, diagnosis, treatment, etc.

## Methods

### Data collection

Data were retrieved and downloaded from PubMed, a biomedical literature database developed by the US National Center for Biotechnology information. Articles from PubMed are indexed with MeSH (Medical Subjects Headings) terms, a set of normalized words that can reflect the content of articles. Based on those MeSH words, the co-word clustering analysis can be performed [7]. In this study, relevant articles were retrieved by searching PubMed without the restriction of language. Retrieval strategy employed was “choroidal neovascularization” [MeSH]. The publication scope was limited from Jan 1st, 2012- May 31st, 2017 and a total of 2366 articles were retrieved. The primary search was conducted by two investigators independently screened these publications based on titles, abstracts and the full text in some cases. The concordance rate between these two investigators was 0.90, indicating a strong agreement [12]. Any discrepancies were discussed until a consensus was reached.

Each publication downloaded from PubMed contained the following items: title, author, institution, country, publication year and MeSH terms. These data were saved as XML format.

### Data extraction and matrix setup

Bibliographic Item Co-Occurrence Matrix Builder (BICOMB) [7], can accurately extract and count the bibliographic information from worldwide databases to generate a co-occurrence matrix, and provide basic data for subsequent statistical analysis. This soft was employed to determine the distribution of the publication year, journals and the frequency ranking of major MeSH terms/MeSH subheadings of the included publications. In addition, the frequency of MeSH terms was recorded and sorted. In this study, MeSH terms, with an occurrence greater than or equal to 20 times, were defined as high-frequency MeSH terms. Thereafter, 44 high-frequency MeSH terms were extracted from the included publications to represent the research hot spots of CNV. According to the co-occurrence situation of these high-frequency MeSH terms in the same article, a MeSH term-source article matrix was built with MeSH terms as the row name and source articles as the column names. The hierarchical cluster analysis was set up based on this MeSH term-source article matrix. Meanwhile, a 44*44 high-frequency MeSH terms co-occurrence matrix was constructed to support further co-word analysis of strategic diagram and centrality description with SNA.

### Clustering analysis of the high frequency MeSH terms

We employed the hierarchical clustering analysis to evaluate the above high-frequency MeSH terms. Average linkage cluster analysis in SPSS 19.0 statistical software was applied to construct clustering relationship dendrogram. These high-frequency MeSH terms were combined according to the similarity degrees. With the help of semantic relationships among the MeSH terms and the content of the representative papers in each cluster, the basic framework of research hot spots of CNV was drawn and analyzed.

### Strategic diagram analysis

A strategic diagram is a two-dimensional space built by plotting themes according to their centrality and density along two axes [13]. The X-axis represents centrality or the external cohesion index, namely the central position of the theme within the overall network. The Y-axis represents density or the internal cohesion index, namely the conceptual development of the theme [14]. Four quadrants were generated with the X- and Y-axis. The above mentioned six categories were then allocated into these four quadrants according to the results of the clustering analysis. Furthermore, excel was used to generate a strategic diagram.

### Social network analysis

The high-frequency MeSH terms co-occurrence matrix was imported into the Ucinet 6.0 (Analytic Technologies Co., Lexington, KY, USA) software, after which the SNA method was used to analyze the themes and knowledge structure of CNV. To visualize the network structure, the MeSH term networks were displayed in two dimensional maps by the software NetDraw2.084. The nodes of the network are the major MeSH terms/MeSH subheadings, and the links represent the co-occurrence frequency of these terms. To understand the structure of the network on CNV, we evaluated the location of these MeSH terms in the network by measuring the degree, betweenness and closeness centralities of each node.

## Results

### Distribution characteristics of relevant literatures

**Fig. 1:**
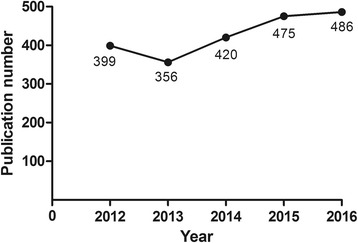
Number of publications on choroidal neovascularization from 2012 to 2016

Based on the search strategy, a total of 2366 publications (Jan. 1st, 2012-May. 31st, 2017) were included in this study. In the past 5 years, researchers were paying increasing attention on CNV. As shown in Fig. 1, the annual publication of articles has gradually increased from 399 in 2012, to 486 in 2016 in the fields of CNV. Altogether, 358 journals have been involved in this field. Table 1 displays the top ten productive journals, which are considered as the core journals in this research area. Among the top ten journals, the top three journals are Retina, Investigative Ophthalmology & Visual Science (IOVS) and Ophthalmology, and these three journals consist more than 20% of the total searched literatures in this field. The US is by far the greatest contributor of ophthalmic researches and institutions from US and England have conducted more than 60% of the researches in this specific area.

**Table 1:** Temporal distribution of publications on CNV in PubMed (2012-May 2017)

Rank	Country	Publications n (%)	Top journal	Publications n (%)	Author (No. of paper)
1	United States	1084 (45.8)	Retina (Philadelphia, Pa.)	226 (9.6)	Querques G (15)
2	England	468 (19.8)	Investigative ophthalmology & visual science	147 (6.2)	Kim JH (15)
3	Germany	153 (6.5)	Ophthalmology	101 (4.3)	Parodi MB (10)
4	Switzerland	123 (5.2)	American journal of ophthalmology	93 (3.9)	Battaglia Parodi M (9)
5	Netherlands	80 (3.4)	The British journal of ophthalmology	74 (3.1)	Wang H (9)
6	New Zealand	68 (2.9)	Graefe’s archive for clinical and experimental ophthalmology	72 (3.0)	Iacono P (9)
7	China	56 (2.4)	PloS one	70 (3.0)	Tan CS (9)
8	Italy	45 (1.9)	Eye (London, England)	49 (2.1)	Chhablani J (8)
9	India	40 (1.7)	Acta ophthalmologica	48 (2.0)	Lee JH (7)
10	France	38 (1.6)	Clinical ophthalmology (Auckland, N.Z.)	46 (1.9)	Cheung CM (7)
Total		2155 (91.1)		926 (39.1)	

### Research hot spots concluded by MeSH term clusters

For the included publications, there were 1983 MeSH terms with a cumulative frequency of 8188 times. As shown in Table 2, the cumulative frequency percentage of the 44 high-frequency MeSH terms accounts for 50.38% (4125/8188) of the total MeSH terms. These MeSH terms could represent the research hot spots on CNV in this past 5 years.

**Table 2:** High-frequency MeSH terms from the included papers on CNV (*n* = 1983)

Rank.	Major MeSH terms/ MeSH subheadings	Frequency	Proportion of frequency (%)	Cumulative percentage (%)
1	Choroidal Neovascularization / drug therapy	498	6.0821	6.0821
2	Angiogenesis Inhibitors / therapeutic use	484	5.9111	11.9932
3	Antibodies, Monoclonal, Humanized / therapeutic use	310	3.7860	15.7792
4	Tomography, Optical Coherence / methods	205	2.5037	18.2829
5	Choroidal Neovascularization / diagnosis	204	2.4915	20.7743
6	Choroidal Neovascularization / pathology	192	2.3449	23.1192
7	Wet Macular Degeneration / drug therapy	138	1.6854	24.8046
8	Macular Degeneration / drug therapy	134	1.6365	26.4411
9	Vascular Endothelial Growth Factor A / antagonists & inhibitors	116	1.4167	27.8578
10	Retinal Pigment Epithelium / pathology	109	1.3312	29.1891
11	Fluorescein Angiography / methods	109	1.3312	30.5203
12	Ranibizumab / therapeutic use	91	1.1114	31.6317
13	Choroidal Neovascularization / etiology	91	1.1114	32.7430
14	Photochemotherapy / methods	90	1.0992	33.8422
15	Recombinant Fusion Proteins / therapeutic use	86	1.0503	34.8925
16	Macular Degeneration / pathology	77	0.9404	35.8329
17	Choroidal Neovascularization / genetics	77	0.9404	36.7733
18	Receptors, Vascular Endothelial Growth Factor / therapeutic use	74	0.9038	37.6771
19	Choroid / blood supply	69	0.8427	38.5198
20	Visual Acuity / physiology	65	0.7938	39.3136
21	Choroidal Neovascularization / metabolism	61	0.7450	40.0586
22	Wet Macular Degeneration / diagnosis	59	0.7206	40.7792
23	Myopia, Degenerative / drug therapy	55	0.6717	41.4509
24	Macular Degeneration / diagnosis	54	0.6595	42.1104
25	Macular Degeneration / genetics	49	0.5984	42.7088
26	Polymorphism, Single Nucleotide	47	0.5740	43.2829
27	Polyps / drug therapy	46	0.5618	43.8447
28	Choroidal Neovascularization / prevention & control	44	0.5374	44.3820
29	Myopia, Degenerative / complications	44	0.5374	44.9194
30	Polyps / diagnosis	41	0.5007	45.4201
31	Retinal Neovascularization / drug therapy	36	0.4397	45.8598
32	Vascular Endothelial Growth Factor A / metabolism	35	0.4275	46.2872
33	Macular Degeneration / complications	35	0.4275	46.7147
34	Bevacizumab / therapeutic use	35	0.4275	47.1422
35	Choroidal Neovascularization / complications	34	0.4152	47.5574
36	Retinal Pigment Epithelium / metabolism	33	0.4030	47.9604
37	Disease Models, Animal	32	0.3908	48.3512
38	Macular Degeneration / metabolism	29	0.3542	48.7054
39	Choroidal Neovascularization / physiopathology	26	0.3175	49.0230
40	Angiogenesis Inhibitors / adverse effects	24	0.2931	49.3161
41	Wet Macular Degeneration / genetics	23	0.2809	49.5970
42	Antibodies, Monoclonal, Humanized / adverse effects	23	0.2809	49.8779
43	Retinal Drusen / diagnosis	21	0.2565	50.1343
44	Myopia, Degenerative / diagnosis	20	0.2443	50.3786

**Fig. 2:**
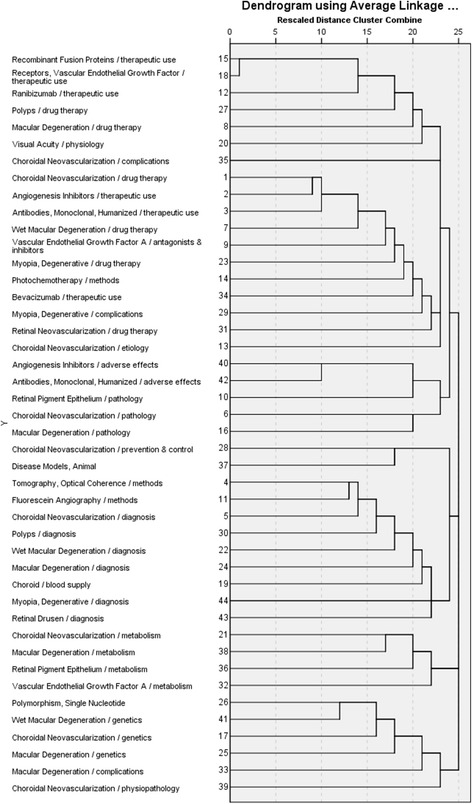
Dendrogram of 44 high-frequency MeSH terms

Based on the hierarchical clustering analysis, the MeSH terms were analyzed and classified into 6 categories (Fig. 2 & Table 3). These categories include: (1) Clinical drug therapy of age-related macular degeneration (AMD), PCV and degenerative myopia, the drugs of which include vascular endothelial growth factor A (VEGFA) inhibitors, and monoclonal antibodies and recombinant fusion proteins; (2) Study on etiology, complications, prevention and control of CNV in animal models; (3) Pathology of CNV and adverse effect of angiogenesis inhibitor; (4) Diagnostic methods, including optical coherence tomography (OCT) and fundus fluorescein angiography (FFA), and diagnostic criteria of CNV, polyps, drusen and degenerative myopia; (5) Metabolism related researches on CNV, retinal pigment epithelium (RPE) and macular degeneration; (6) Genetics related researches on macular degeneration and degenerative myopia. These six categories could represent the major research topics in these 5 years.

**Table 3:** Cluster analysis of MeSH terms

Cluster	Number of MeSH terms^a^	Cluster analysis
1	15,18, 12,27,8,20,1,2,3,7,9,23,14,34,29,31	Clinical drug therapy of AMD, PCV and degenerative myopia
2	35,13,28,37	Study on etiology, complications, prevention and control of CNV in animal models
3	40,42,10,6,16	Pathology of CNV and adverse effect of angiogenesis inhibitor
4	4,11,5,30,22,24,19,44,43	Diagnostic criteria and methods of CNV, polyps, drusen and degenerative myopia.
5	21,38,36,32	Metabolism related researches on CNV
6	26,41,17,25,33,39	Genetics related researches on macular degeneration and degenerative myopia

^a^Represents the serial number of high-frequency MeSH terms

### Strategy diagram for CNV

**Fig. 3:**
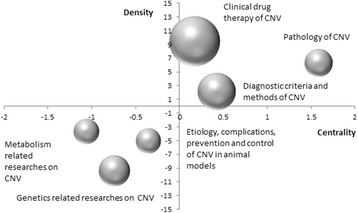
Strategic diagram for choroidal neovascularization

Motor-themes are those with both strong centrality and high density as shown in Quadrant I (upper-right). Specialized themes are those in Quadrant II (upper-left) and are defined as those with inadequate external interactions but high density. Quandrant III (lower-left) contains themes with weak density and inadequate centrality, and these themes are usually considered to be either appearing or vanishing. The last quadrant, Quandrant IV (lower-right), contains themes with strong centrality but lacking of internal maturation. [13]. In strategic diagrams, themes are represented by spheres of different areas, which are organized in different quadrants according to their internal and external cohesion (density and centrality, respectively). As shown in Fig. 3, the area of the spheres is proportional to the number of high-frequency MeSH terms. Cluster No. 1, 3 and 4 locate in Quadrant I, representing that researches on clinical drug therapy, diagnostic criteria and methods, as well as the pathology of CNV are in the core status with high density and centrality. Cluster No. 2, 5 and 6 locate in Quadrant III, indicating that researches on etiology, complications, prevention and control of CNV in animal models, as well as metabolism and genetics related studies on CNV are not mature, namely on the edge of the research field.

### Social network analysis of CNV

An SNA is presented in Tables 4 and 5, and degree, betweenness and closeness centrality are applied to analyze the SNA network structure.

**Table 4:** Individual centrality of CNV research from 2012-May 2017

No.	Major MeSH terms / MeSH subheadings	Degree	Betweenness	Closeness
1	Choroidal Neovascularization / drug therapy	1259	22.29	39.50
2	Angiogenesis Inhibitors / therapeutic use	1374	38.64	41.50
3	Antibodies, Monoclonal, Humanized / therapeutic use	818	28.90	40.00
4	Tomography, Optical Coherence / methods	462	18.61	38.50
5	Choroidal Neovascularization / diagnosis	437	12.57	37.00
6	Choroidal Neovascularization / pathology	331	41.27	41.50
7	Wet Macular Degeneration / drug therapy	404	13.67	36.50
8	Macular Degeneration / drug therapy	336	18.31	36.00
9	Vascular Endothelial Growth Factor A / antagonists & inhibitors	350	23.07	39.00
10	Retinal Pigment Epithelium / pathology	242	24.35	38.50
11	Fluorescein Angiography / methods	272	9.26	34.50
12	Ranibizumab / therapeutic use	307	8.82	36.00
13	Choroidal Neovascularization / etiology	97	11.03	36.00
14	Photochemotherapy / methods	219	4.74	34.50
15	Recombinant Fusion Proteins / therapeutic use	322	7.36	36.00
16	Macular Degeneration / pathology	124	29.47	38.00
17	Choroidal Neovascularization / genetics	96	8.05	32.50
18	Receptors, Vascular Endothelial Growth Factor / therapeutic use	292	5.88	35.50
19	Choroid / blood supply	140	18.72	38.00
20	Visual Acuity / physiology	186	6.62	35.50
21	Choroidal Neovascularization / metabolism	57	1.73	27.00
22	Wet Macular Degeneration / diagnosis	135	5.98	33.00
23	Myopia, Degenerative / drug therapy	188	5.38	33.50
24	Macular Degeneration / diagnosis	113	5.46	33.00
25	Macular Degeneration / genetics	65	2.26	30.00
26	Polymorphism, Single Nucleotide	87	3.84	30.50
27	Polyps / drug therapy	173	0.16	29.00
28	Choroidal Neovascularization / prevention & control	40	5.44	30.50
29	Myopia, Degenerative / complications	116	3.11	32.00
30	Polyps / diagnosis	110	0.59	26.83
31	Retinal Neovascularization / drug therapy	90	1.59	30.00
32	Vascular Endothelial Growth Factor A / metabolism	54	14.20	33.50
33	Macular Degeneration / complications	63	4.65	32.50
34	Bevacizumab / therapeutic use	123	2.83	32.50
35	Choroidal Neovascularization / complications	48	3.97	33.00
36	Retinal Pigment Epithelium / metabolism	39	4.63	29.00
37	Disease Models, Animal	44	0.92	28.50
38	Macular Degeneration / metabolism	26	0.79	24.67
39	Choroidal Neovascularization / physiopathology	35	6.35	30.50
40	Angiogenesis Inhibitors / adverse effects	48	0.34	27.50
41	Wet Macular Degeneration / genetics	38	0.12	25.33
42	Antibodies, Monoclonal, Humanized / adverse effects	62	2.52	30.00
43	Retinal Drusen / diagnosis	38	1.04	27.50
44	Myopia, Degenerative / diagnosis	44	1.50	28.00

**Table 5:** Descriptive statistics for centrality measure about CNV

Centralization	Mean ± SD	Min	Max	Network centralization
Degree	225.091 ± 284.624	26	1374	8.791%
Betweenness	9.795 ± 10.292	0.118	41.269	3.57%
Closeness	33.235 ± 4.393	24.667	41.500	39.80%

In the network of “CNV”, 14 MeSH terms are shown to have a degree centrality more than the mean value of 225.091, and the top ten high-frequency MeSH terms are also included. Among these ten high-frequency MeSH terms, “Angiogenesis Inhibitors/therapeutic use” displays the highest degree centrality of 1374.

The top two betweenness centrality values listed in Table 4 are 41.27 and 38.64, representing “Choroidal Neovascularization/pathology” and “Angiogenesis Inhibitors/therapeutic use”, respectively. These two MeSH terms have the strongest mediating role in the network. As shown in Table 5, the mean betweenness centralization is 9.795. MeSH terms “Macular degeneration/pathology”, “choroid/blood supply”, and “Vascular Endothelial Growth Factor A/metabolism” show relative higher betweenness centrality value of 29.47, 18.72 and 14.203, respectively. Whereas, the degree centralities of these MeSH terms are 124, 140 and 54, respectively, which are far lower than their mean degree centrality value of 225.091.

As shown in Table 4, MeSH terms “Choroidal Neovascularization/pathology” and “Angiogenesis Inhibitors/therapeutic use” both present the top two closeness centrality value of 41.5.

**Fig. 4:**
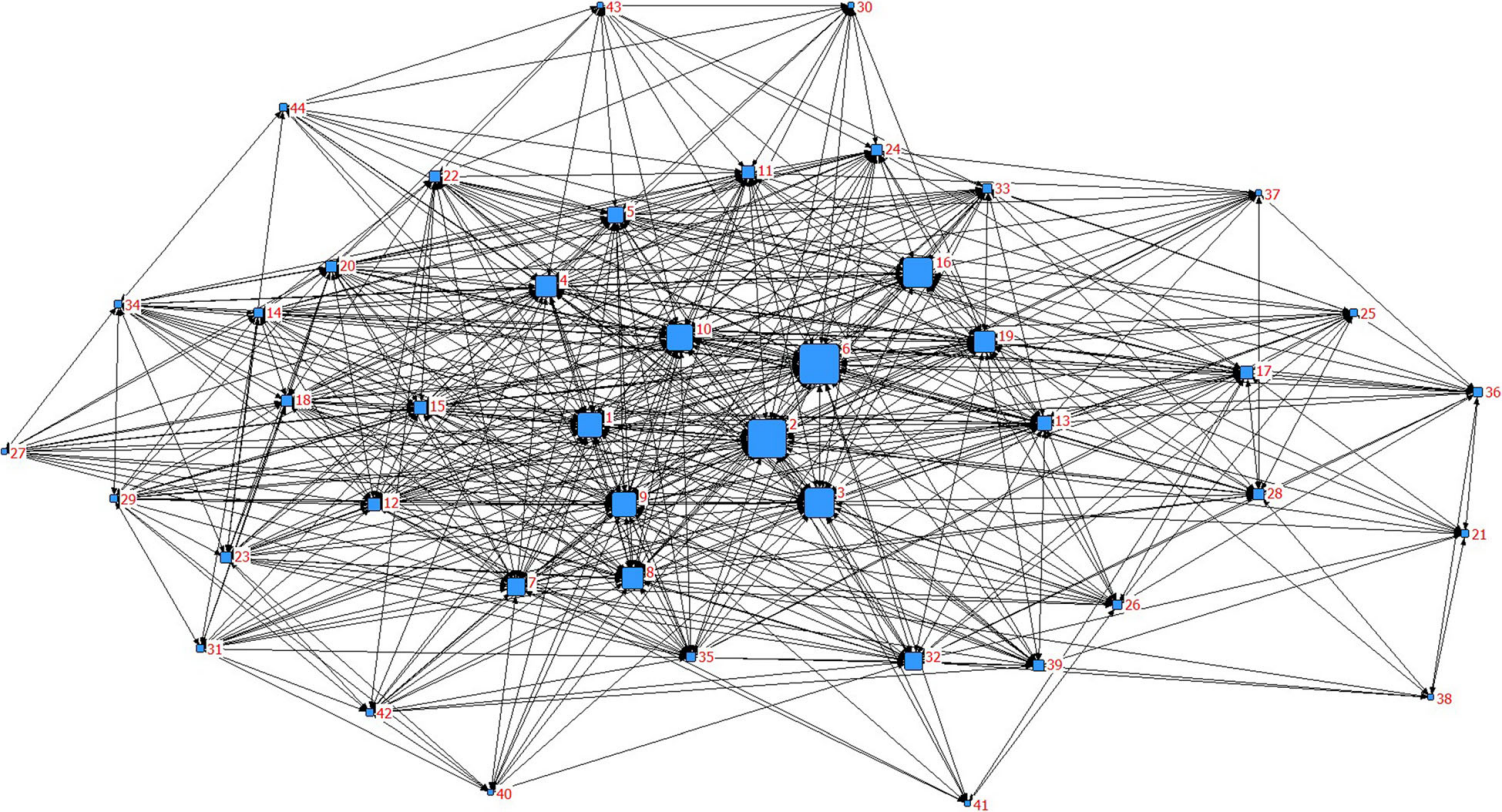
SNA of 44 high-frequency MeSH terms

To understand easier, SNA was drawn based on the betweenness centrality. As seen in Fig. 4, the size of nodes indicates the MeSH terms betweenness centrality and the thickness of lines represents the co-occurrence frequency.

## Discussion

MeSH terms can reveal the most accurate content of these literatures and a large collection of MeSH terms can reflect the research status of the discipline and trends. Vision Disorders, Glaucoma, Diabetic Retinopathy, Macular Degeneration, and Cataract are the most frequent MeSH terms related to eye diseases [15]. Through the statistical analysis with BICOMB software, the distribution characteristics of the literatures on “choroidal neovascularization” [MeSH] in recent 5 years maintains a fluctuating increase with a slight decline in 2013. Moreover, our analysis revealed that the US and England are the biggest contributors in the research of CNV, which is consistent with the bibliometric results of other fields [16–18]. This could be explained by the reason that English is the first language in these two countries.

In order to systematically examine the fundamental knowledge structure of CNV, this study integrated co-word analysis and SNA based on Bibliometry. According to the co-word analysis, closely related MeSH terms can be gathered and form clusters. Cluster 1 relates to the drug therapy of wet macular degeneration, degenerative myopia and PCV. Drug therapies mainly focus on monoclonal antibody and recombinant fusion proteins. In the angiogenesis process, VEGF-A stimulates the growth of the abnormal blood vessels. Medications that can block this protein include ranibizumab, bevacizumab and aflibercept. Ranibizumab is a humanized, monoclonal, VEGF-specific antibody fragment that can prevent it from binding to its receptor, thus inhibiting angiogenesis. Bevacizumab is a VEGF-specific full-length humanized monoclonal antibody. The effectiveness and safety of these two medications for the treatment in neovascular AMD have been analyzed to be similar [19]. Aflibercept is a novel fusion protein which binds to VEGF-A, VEGF-B and placental growth factor (PIGF). Since it possesses stronger binding affinity for VEGF than the previous mentioned two medications, it allows longer intervals between treatments [20]. Cluster 3 relates to the pathology of CNV, adverse effects of angiogenesis inhibitors and monoclonal antibodies. Ocular neovascularization includes retinal neovascularization and subretinal or choroidal neovascularization. DR and retinal vein occlusions are the most prevalent ischemic retinopathies relate to retinal neovascularization. Subretinal or choroidal neovascularization occurs in diseases of the outer retina and Bruch’s membrane, the most prevalent of which is AMD [21]. CNV is a common pathological process in a heterogeneous variety of chorioretinal disease. Any pathologic changes that involve RPE and damages to Bruch’s membrane can be complicated by CNV [22]. Experimental evidences showed that pathogenesis of CNV involve the angiogenesis of vascular component and inflammation of extravascular component [23]. Today, intravitreal VEGF inhibitors are the mainstay of treatment worldwide. It also covers emerging therapies including radiation, latest generation anti-VEGF agents and combination therapies [24]. The most frequently reported ocular adverse events after intravitreal injection are inflammation and increased intraocular pressure [19]. Cluster 4 relates to the diagnostic methods and criteria of CNV in PCV, wet macular degeneration and degenerative myopia. With the help of diagnostic imaging methods such as OCT and FFA, it is feasible to detect subtle exudation in some individuals who have experienced a recent change in visual acuity [19]. By means of SD OCT (spectral domain optical coherence tomography), outer retinal tubulations (ORTs) mainly present circular or ovoid shape and hyperreflective material can also be observed in the border. ORTs are the common symbols in eyes with CNV and geographic atrophy [25]. The emergence of these structures usually indicates the next anti-VEGF drugs application should be considered. However, their non-detection means repeated intravitreal injection of anti-VEGF drugs is temporarily unneeded [26]. These three clusters (Cluster 1, 3, 4) located in Quadrant I, which demonstrates these research hotspots are centralized and well developed.

Cluster 2 relates to etiology, prevention and complications of CNV in animal models. In the eye, many diseases involve angiogenesis, including CNV in AMD and retinal neovascularization in DR and retinopathy of prematurity (ROP) [27]. Numerous studies in mouse models have helped to elucidate the molecular pathogenesis underlying retinal, subretinal and choroidal neovascularization. Currently, three animal models are commonly recognized to study ocular neovascularization in diseases of AMD, proliferative diabetic retinopathy (PDR) and ROP [2]. The most commonly used mouse model for analyzing AMD caused by CNV formation is the laser-induced CNV model, even though without the existence of macula in mice. In this model, due to the lesions caused by laser photocoagulation, new blood vessels are formed from choroid to subretinal space, presenting major characteristics of wet AMD. PDR is a most typical form of retinal neovascularization causing vision loss in patients. Factors including persistent high blood pressure, hyperglycemia and hypoxia cause initial damages to retinal capillaries. Subsequently, abnormal neovascularization is established both along the retina, as well as inside the vitreous. The most typical animal model for PDR can be represented by streptozotocin (STZ) intraperitoneal administration in mice. Oxygen-induced retinopathy (OIR) is the typical animal model for studying the ROP. This model is based on the disease mechanisms of the ROP and is used to investigate and analyze the abnormal neovascularization caused by ischemia [2]. Cluster 5 relates to metabolism on CNV. Oxidative stress typically causes endothelial cell dysfunction, pericyte apoptosis and angiogenesis, which further result in retinopathy [28]. Angiogenesis is a complex process whereby interactions between stimulatory and inhibitory factors result in new blood vessel formation. Antiangiogenic therapies function either by blocking stimulatory factors or by promoting inhibitory factors, thus, disrupting the formation of new vessels. Cluster 6 relates to genetics researches on macular degeneration. Gene therapy is a mean of treating diseases and disorders caused by gene abnormal expression through the insertion of specific genes in vivo [29]. Recently, the clustered regularly interspaced short palindromic repeats (CRISPR)/CRISPR-associated protein 9 (Cas9) system has been developed as a novel genome-editing tool in numerous medical aspects including ocular diseases [30]. Currently, limitations of gene therapy include inefficient and unsustainable target gene expression inside the cells, and concerns of using viral vectors for target gene delivery. However, it is believed to be an evolving technique with comprehensive applications soon in the future. These three clusters located in Quadrant III indicate that these existing research hotspots are peripheral and undeveloped, and further study on these themes are recommended in the future.

The SNA result shows that the top ten high-frequency MeSH terms also possess relatively high degree centrality. According to the measurement of the degree centrality, we conclude that MeSH terms such as “Angiogenesis Inhibitors/therapeutic use” have the most number of direct connections with other components and lead the development in the field of retinopathy. As for the betweenness centrality in this study, “Choroidal Neovascularization/pathology” and “Angiogenesis Inhibitors/therapeutic use” are in the hub position of the whole network, which represent these dominant components have the greatest potential on controlling the co-occurrence of other components. However, hotspots on genetics and the metabolism of CNV are along the edge of the network. This phenomenon indicates that pathology and treatment of CNV put researches on retinopathy in motion, whereas the researches about genetics and metabolism of CNV are the emerging field. The MeSH terms “Macular degeneration/pathology”, “choroid/blood supply”, and “Vascular Endothelial Growth Factor A/metabolism” show relative high betweenness centralities, whereas the degree centralities are lower than the mean value. This demonstrates that although these components do not show distinct direct relations with other nodes, they occupy the intermediary position and are of great importance in maintaining the stability of the whole network.

## Conclusions

In conclusion, hierarchical clustering analysis and strategic diagram can be used to demonstrate the thematic structure of a specific field and estimate the maturing status of each cluster, respectively. However, these two methods fail to decipher the central MeSH term and reveal the relationship of each component. SNA makes up the deficiency of the aforementioned methods and depicts the relationships among high-frequency MeSH terms in a system. The size of nodes and the thickness of lines represent the position of the MeSH term in the whole network. The characteristics of these methods are summarized in Supplementary Data 1.

Our study integrated the strategic diagram and SNA based on the co-word analysis of MeSH terms on CNV. Researches on the drug therapy and pathology changes are in the core status, whereas studies on metabolism and genetics are the emerging topics. Although our study could provide some hints for researches in choosing the research topics, the results of our analyses are affected by some methodological limitations that should be considered. Firstly, the majority of journals and literatures included in PubMed are in English thus the non-inclusion of all national journals may also influence the result to some extent. Secondly, many papers could be published in the subspecialty while having little impact on the field. Conversely, some elite journals are acknowledged to carry articles of generally high quality and therefore be relatively selective in the articles they publish. Thus, the papers may contribute different weight in the knowledge structure. Thirdly, the co-word analysis is based on high-frequency MeSH terms. Thus, the amount of high-frequency MeSH terms might have some influence on the clustering analysis result, and the new emerging topics with low attention may not have been included. Therefore, analyses combining multiple databases and new emerging topics should be conducted in the future studies.

## Abbreviations

BICOMB: Bibliographic item co-occurrence matrix builder
CNV: Choroidal neovascularization
MeSH: Medical subject headings
SNA: Social network analysis
